# African trypanosomiasis

**DOI:** 10.1111/j.1365-3024.2011.01302.x

**Published:** 2011-08

**Authors:** L J Morrison, A MacLeod

**Affiliations:** 1 Wellcome Trust Centre for Molecular Parasitology, Institute of Infection, Immunity and Inflammation, College of Medical, Veterinary and Life Sciences, University of GlasgowGlasgow, UK; 2 Wellcome Trust Centre for Molecular Parasitology, Institute of Biodiversity, Animal Health and Comparative Medicine, College of Medical, Veterinary and Life Sciences, University of GlasgowGlasgow, UK

African trypanosomiasis remains a serious health concern across large areas of sub-Saharan Africa, despite several decades of research. As well as causing sleeping sickness in humans, trypanosomes are responsible for significant disease in livestock. The combination of human and livestock disease makes these parasites a serious impediment to agricultural and economic advancement in the affected areas, an impact that combines with the obvious health concerns to create a problem that seriously impinges at several levels upon human wellbeing. Despite this seemingly pessimistic summary, significant strides have been made in our understanding of the interactions between the host and the parasite that drive disease and shape the course of infections. This volume will attempt to summarise recent significant findings, outlining the holistic way in which our understanding is progressing, encompassing human and livestock disease, disease determinants in both the host and the parasite, and the potential that is arising for novel therapeutics from the often neglected member of the lifecycle, the tsetse fly. Given the emerging drug resistance in both human and livestock licensed trypanocidal drugs and the fact that a vaccine is unlikely for African trypanosomes, understanding the disease process and the key host–parasite interactions that influence this is an obvious route by which novel interventions may be developed.

An obvious first step in this process is to comprehend what occurs in the mammalian host when it is infected with trypanosomes. A crucial component that has contributed significantly to our knowledge of what influences the outcome of trypanosome infection has been the development over the decades of a multi-faceted rodent model of disease. Although not the ‘natural host’ of trypanosome infections, Stefan Magez and Guy Caljon (1) review key insights that the rodent model has enabled, and which would have been extremely difficult if not impossible without this tractable system. These include how the trypanosome interacts and is affected by both the humoral and innate immune responses, how human-infective trypanosomes are able to avoid lysis by human serum (which has the innate ability to kill all other species of African trypanosome – this work was first pioneered in the mouse), and the influence of tsetse saliva on the immune response at the bite site. Boniface Namangala (2) expands on these themes and outlines the impact of trypanosome infection upon lymphocyte responses. While the rodent model has undoubtedly hugely enriched our knowledge of immunopathogenesis in trypanosomiasis, complementary studies in natural hosts are a key component in confirming translation of any findings. Bruno Bucheton, Annette MacLeod and Vincent Jamonneau (3) describe recent data from sleeping sickness patients. These findings indicate that the classically described progression of *Trypanosoma brucei gambiense* infection leading through Stage 1 disease (haemolymphatic) to Stage 2 symptoms (neurological) and then death is too simplistic. They describe the diversity of disease presentation and progression observed, discuss the probability of ‘trypanotolerance’ (whereby the genetics of the host plays a role in susceptibility or otherwise to trypanosome infections, i.e., trypanotolerant hosts remain infected but do not display the serious clinical signs displayed by susceptible counterparts) in humans, and review what is currently known about possible host factors that may contribute to this.

While a significant amount of research has much improved our understanding from the mammalian host's perspective, a significant factor in disease pathology is obviously supplied by the parasite. Genetic variation in the host, as outlined above, has been the foundation of a formidable body of work that has aimed to identify host genes and gene variants (in mice and cattle) that determine the severity of clinical signs during trypanosome infection. Liam Morrison (4) describes recent approaches that have used genetic variation in *T. brucei* as a basis for identifying parasite genes and gene products that influence the disease phenotype in the host. Although this parasite-derived variation in host phenotype has long been recognised, for example, the classical description of the human disease caused by the subspecies of *T. brucei* as chronic or acute depending on the infecting subspecies, only relatively recently has the qualitative evaluation of the contribution of parasite genetic variation emerged as a focus of research. It is clear that for a holistic understanding of the relative contribution of the parasite and host to disease outcome, this is an aspect that will be important in the future. This theme is extended by Peter Van den Bossche, Simbarasha Chitanga, Justin Masumu, Tanguy Marcotty and Vincent Delespaux (5), to the most important trypanosome with respect to livestock morbidity and mortality in sub-Saharan Africa, *Trypanosoma congolense*. They outline their previous work that describes virulence variation in field isolates of *T. congolense* and revisit this with new analysis that contextualises the epidemiological influences and impact of trypanosomes and virulence. This is done by comparing the virulence of strains from both sylvatic and domestic life cycles, and the authors suggest that the relative selective forces of each system will influence the virulence of the parasite.

Finally, John Harrington (6) describes how antimicrobial peptides, derived from a variety of biological sources, may be potentially exploited to develop novel therapies for trypanosome infections in the mammalian host. The role that these small molecules play in the tsetse innate immune response against trypanosomes is described. Current knowledge of the identity and diversity of antimicrobial peptides is summarized, and recent studies that have assessed the trypanocidal ability of synthetic and natural antimicrobial peptides (from a variety of mammals as well as fungi), in addition to that of some unconventional peptides, are discussed. Antimicrobial peptides represent an example of how a relatively neglected area can represent a hitherto untapped resource for novel approaches.

The collection of articles highlights much of the progress that has been made in terms of understanding the disease process from the perspective of the mammalian host, the tsetse host and the parasite. They also make clear the power and the necessity of approaches that improve our knowledge base for all three components of the life cycle. It is also evident that there are significant challenges to be overcome, in terms of not only understanding fully the basic and key host–parasite interactions that influence the course of disease but also exploiting any findings to reduce the burden of trypanosomiasis at an applied level.

The editors would like to thank all of the authors for their contribution to this volume, and Mrs Emma Missen, the co-ordinating editor. Our last note is to acknowledge the sad passing of Peter Van den Bossche during the preparation of this issue, who has contributed so much to the understanding of trypanosome biology.

## References

[1] Magez S, Caljon G (2011). Mouse models for pathogenic African trypanosomes: unravelling the immunology of host-parasite-vector interactions. Parasite Immunol.

[2] Namangala B (2011). How the African trypanosomes evade host immune killing. Parasite Immunol.

[3] Bucheton B, MacLeod A, Jamonneau V (2011). Human host determinants influencing the outcome of *Trypanosoma brucei gambiense* infections. Parasite Immunol.

[4] Morrison LJ (2011). Parasite-driven pathogenesis in *Trypanosoma brucei* infections. Parasite Immunol.

[5] Van den Bossche P, Chitanga S, Masuma J, Marcotty T, Delespaux V (2011). Virulence in *Trypanosoma congolense* Savannah subgroup. A comparison between strains and transmission cycles. Parasite Immunol.

[6] Harrington JM (2011). Antimicrobial peptide killing of African trypanosomes. Parasite Immunol.

